# Induction Chemotherapy Prior to Endoscopic Resection of Alveolar Rhabdomyosarcoma

**DOI:** 10.7759/cureus.48761

**Published:** 2023-11-13

**Authors:** Daniel H Lofgren, Benjamin Gillette, Brandon B Knight, Eric Succar

**Affiliations:** 1 Otolaryngology - Head and Neck Surgery, McLaren Oakland Hospital, Pontiac, USA; 2 Otolaryngology - Head and Neck Surgery, St. Joseph Mercy Oakland Hospital, Pontiac, USA

**Keywords:** head and neck rhabdomyosarcoma, post-induction chemotherapy, proton beam radiotherapy, functional endoscopic sinus surgery (fess), alveolar rhabdomyosarcoma

## Abstract

Head and neck rhabdomyosarcoma (HNRMS) is a rare type of soft tissue tumor that affects both adults and children with an overall incidence of 0.041 per 100,000 people. Adults make up approximately 31.2% of all HNRMS diagnoses and have an overall survival rate between 20% and 40%. We present a case of a 46-year-old male who initially presented with nasal congestion and vision changes. Maxillofacial computed tomography and magnetic resonance imaging of the brain showed involvement of the orbital apex, abutment of the planum sphenoidale, and extension to the foramen rotundum (FR). Nasal endoscopy with biopsy confirmed the diagnosis of T2aN0M0 parameningeal HNRMS. The patient underwent induction chemotherapy, followed by endoscopic resection, which resulted in negative intraoperative margins. Subsequently, he underwent adjuvant concurrent chemotherapy and proton beam radiation after positive microscopic positive margins were found on the optic nerve. The patient did not experience any significant complications, and he is currently without radiographic or clinical recurrence 18 months after the treatment. He was able to maintain his vision throughout the treatment. In adults, HNRMS is usually treated with chemoradiotherapy based on pediatric protocols, since there are limited data available for adult treatment protocols and outcomes. Although surgery has been associated with positive outcomes in adult patients, there are no previous reports of its use with either neoadjuvant or adjuvant treatment. This type of treatment protocol has never been described for adult HNRMS. We hope that our report can add more data to the growing body of literature on HNRMS treatment protocols.

## Introduction

Rhabdomyosarcoma (RMS) is a malignant soft tissue tumor composed of immature mesenchymal cells with the potential to differentiate into striated muscle tissue. It is a rare tumor that accounts for only 350 cases annually, yet it comprises about half of soft-tissue sarcomas in children and adolescents [1]. Approximately 35-40% of pediatric RMS occurs in the head and neck region, compared with 33% in adults. In total, RMS represents only 2-5% of soft-tissue tumors in adults [2-4].

Head and neck RMS (HNRMS) has been reported to the Surveillance, Epidemiology, and End Results (SEER) registry 558 times from 1973 to 2007, with an overall incidence of 0.041 cases per 100,000 population [2]. Of all cases, adults make up approximately 31.2% of all diagnoses of HNRMS. Although there is no known gender predilection, it has the propensity to affect white patients more often than other races (77.1% White vs. 13.8% Black vs. 8.4% Others) [2]. HNRMS can be divided into three subtypes based on location with the following frequencies: orbital (25.6%), parameningeal (infratemporal fossa, pterygopalatine fossa, ear, mastoid, nasal cavity, paranasal sinuses) (44.4%), and nonorbital non-parameningeal (tongue, parotid, palate, all other head and neck sites) (29.9%) [2,4]. Three primary histologic subtypes have been identified, namely, pleomorphic, embryonal, and alveolar, with embryonal having a better five-year relative survival of 72.2% compared with alveolar at 44.1% [2].

Here, we present a 46-year-old male with a T2aN0M0 parameningeal HNRMS with involvement of the orbital apex, abutment of the planum sphenoidale, and extension to the foramen rotundum (FR) who underwent induction chemotherapy, followed by endoscopic resection and then by adjuvant concurrent chemotherapy and proton beam radiation. The treatment course was modeled after pediatric protocols for similar parameningeal RMS. To the authors’ knowledge, there are no previous reports of this type of operative and post-operative treatment regimen for parameningeal RMS in an adult, and the patient is currently without radiographic or clinical recurrence 18 months out and has maintained his vision.

## Case presentation

**Figure 1:**
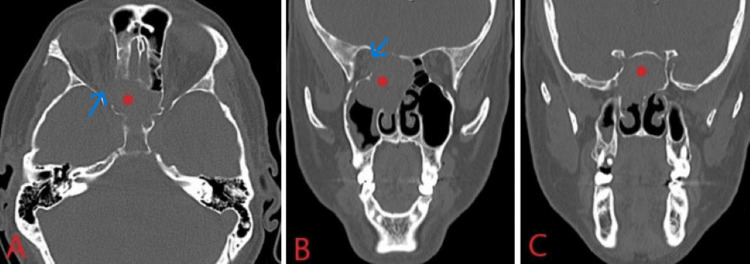
CT maxillofacial without contrast (A), coronal (B), and axial planes (C) in a bone window, showing 4.5 cm x 4.1 cm x 4.0 cm soft tissue mass (red dot) centered in the right ethmoid air cells involving the right sphenopalatine foramen, right olfactory recess, bilateral sphenoid sinuses, and right maxillary sinus. Noted bony destruction of the medial orbital apex and abutment of the right optic nerve (blue arrow), thinning of the right planum sphenoidale and posterior medial right orbital wall, and enlargement of the right maxillary osteomeatal complex (OMC) and posterior fontanelle.

**Figure 2:**
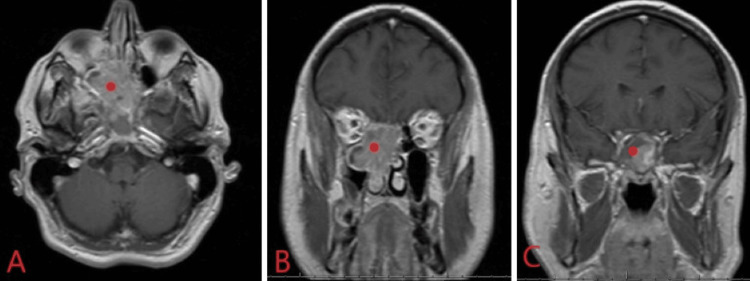
T1 with contrast MRI brain in axial (A) and coronal (B,C) planes showing an enhancing, expansile mass of the right nasal cavity (red dot) extending from the right ethmoid sinus to the bilateral sphenoid sinuses and right anterior skull base, with extension of the mass into the right orbital apex.

A 46-year-old male presented to an outside hospital emergency department with five days of progressively worsening diplopia and nasal congestion. Ophthalmology evaluation noted right papilledema. Non-contrast maxillofacial CT showed a 4.5 cm x 4.1 cm x 4.0 cm soft tissue mass centered in the right ethmoid sinus involving both ethmoid sinuses, both sphenoid sinuses, and the right sphenopalatine foramen with thinning of the planum sphenoidale and erosion into the right orbital apex (Figure 1). MRI of the brain/orbits redemonstrated the expansile mass of the right ethmoid sinus with involvement of the PPF, extension along the planum sphenoidale, and extension through the orbital apex (Figure 2). Otolaryngology was consulted and recommended an urgent nasal biopsy within the week.

The patient underwent an endoscopic image-guided biopsy. The frozen section was not definitive. Permanent histological analysis revealed a small, round, blue-cell tumor, with immunohistochemistry that was positive for desmin, myoD1, and myogenin consistent with RMS. Confirmatory testing with reverse transcription polymerase chain reaction (RT-PCR) was positive for PAX/FOX01 translocation, confirming the alveolar subtype RMS.

After tissue diagnosis, a positron emission tomography (PET) scan redemonstrated the mass centered in the right ethmoid cavity with a maximum standard uptake value (SUV) of 8.6. There was also nonspecific borderline activity of bilateral level 2 lymph nodes with an SUV of 2.8, but no clinical cervical lymphadenopathy on physical exam.

**Figure 3:**
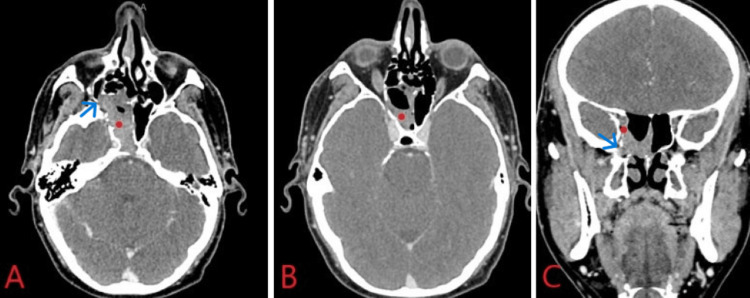
CT scan of sinuses in the axial (A, B) and coronal (C) planes after completion of induction chemotherapy. Noted extension of the mass to the PPF (blue arrow), with a lack of regression from the right maxillary and sphenoid sinuses (red dot).

**Figure 4:**
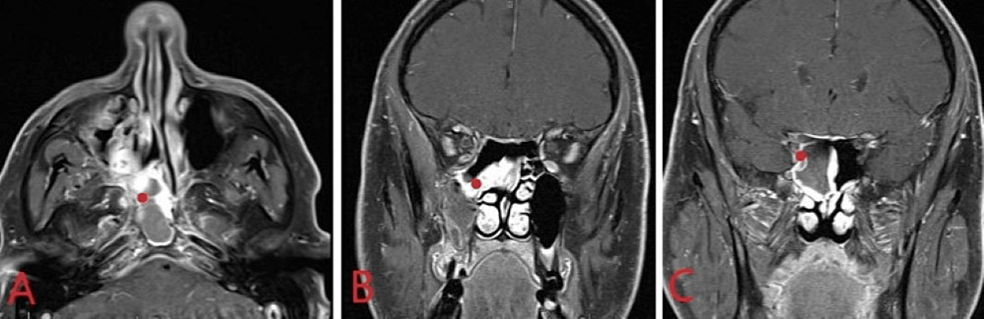
T1 fat-suppressed MRI brain in the axial (A) and coronal (B, C) planes post-induction chemotherapy. Resolution of the mass from the right orbital apex and continued involvement of the right sphenoid sinus laterally and right maxillary sinus anteriorly (red dot).

The patient was presented at a multidisciplinary tumor board as a T2aN0M0 alveolar RMS of the right ethmoid sinus. The tumor board recommendation was for induction neoadjuvant chemotherapy, followed by endoscopic resection and then adjuvant concurrent chemotherapy and proton beam radiation therapy. The patient was strongly opposed to an orbital exenteration. It was felt that this induction chemotherapy (IC) technique, modeled after pediatric protocols, would help facilitate achieving surgical clear margins or at least only microscopic disease at the orbital apex to be followed by adjuvant concurrent chemoradiation therapy. The combination of vincristine, actinomycin D, and cyclophosphamide was chosen according to the D9803 Children’s Oncology Group (COG) protocol. Upon completion of four cycles of induction, both post-treatment CT of the sinuses (Figure 3) and MRI brain (Figure 4) revealed resolution within the orbital apex with continued involvement of the right ethmoid sinus, lateral margin of the right sphenoid sinus, the superior margin of the maxillary sinus, and persistent involvement of the pterygopalatine fossa (PPF).

One month after completion of IC, image-guided endoscopic resection, including a right medial maxillectomy, bilateral ethmoidectomy, bilateral sphenoidotomy, right frontal sinusotomy, near total septectomy, extended right PPF dissection, and right orbital/optic nerve decompression, was performed. Final intraoperative frozen section margins of the posterior periorbita, posterior septum, lateral sphenoid wall/optic nerve sheath, ethmoid/sphenoid skull base, and PPF/vidian canal were negative. Vision remained intact after surgery and no cerebrospinal fluid leak was encountered. Final pathology from the resection again revealed parameningeal alveolar RMS, FOX01 translocation positive. Of note, the lateral sphenoid wall/optic nerve sheath did show evidence of microscopic disease on the permanent section. Four weeks after recovery from surgery, the patient started concurrent chemoradiotherapy (CRT), which included vincristine, cyclophosphamide, actinomycin D, and 23 fractions of proton beam therapy with a total dose of 41.4 Gy.

**Figure 5:**
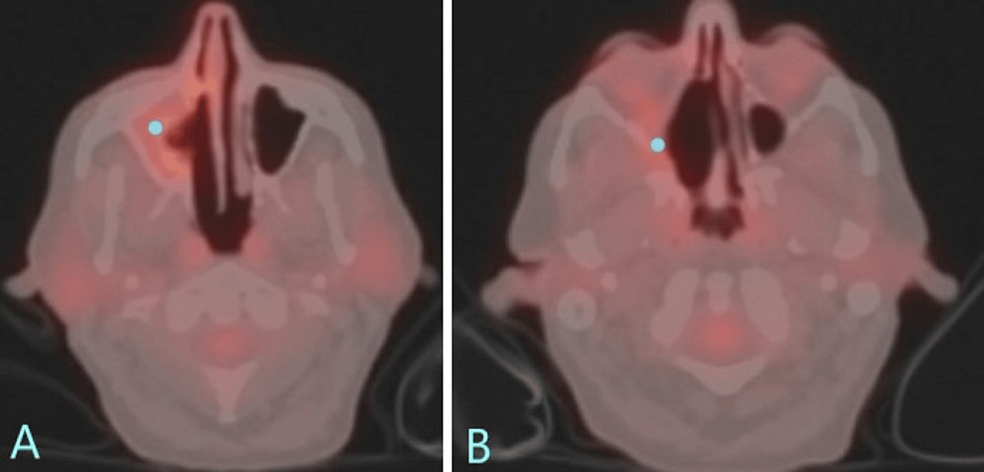
A three-month post-treatment PET-CT axial plane (A, B). Interval right frontoethmoidectomy and bilateral sphenoidectomy with tumor resection noted. A mild amount of mucosal thickening of the right maxillary sinus (blue dot), which was mildly FDG avid, with an SUV 3.4.

**Figure 6:**
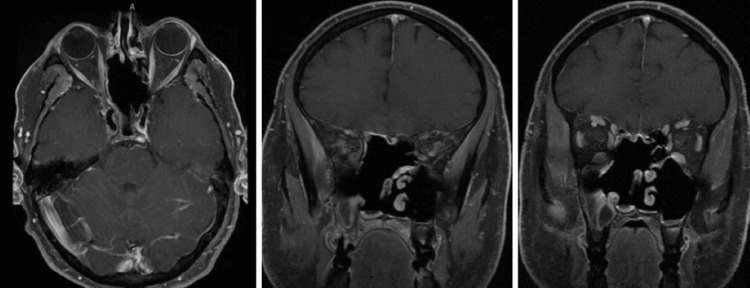
Eleven months post-treatment T1 fat-suppressed post-contrast MRI brain/orbit axial (A) and coronal planes (B, C). Extensive postsurgical changes with no definite signs of residual or recurrent tumor were noted.

Three-month post-treatment PET-CT (Figure 5) revealed a mild amount of mucosal thickening of the right maxillary sinus, which was mildly FDG avid with an SUV of 3.4, compared with his pre-treatment SUV of 8.6, likely representing inflammatory changes rather than residual disease. The most recent MRI (Figure 6) did not show any definite signs of recurrence. The patient is currently 18 months out from completion of CRT and is being monitored at three-month intervals with serial nasal endoscopies and repeat imaging with no signs of recurrence.

## Discussion

RMS is a malignant tumor composed of immature mesenchymal cells with the potential to differentiate into striated muscle tissue and typically presents with small blue round cells on histological examination [5]. The two most common histologic subtypes of RMS are embryonal and alveolar, with embryonal presenting more commonly in children and alveolar more commonly in adults, and immunohistochemical markers are the most reliable way to differentiate between the two [2,5,6]. Myogenin, myoD1, and desmin are more specific to alveolar RMS compared with embryonal, and the presence of PAX/FOX01 translocation favors the alveolar subtype, as seen in our patient [1,5-7].

Overall, RMS treatment relies on the TNM classification, disease site, FOX01 translocation factor presence, and clinical group findings. Due to the rare occurrence of RMS in adults, the prognosis is based on data identified by the Intergroup Rhabdomyosarcoma Study Group Outcomes I and II, which included only those diagnosed in the first two decades of life [8-10]. The data gathered from these studies incorporated tumor spread at diagnosis and the amount remaining after the initial intervention [4,8-10]. Although these prognostic groups were based on pediatric patients, adult RMS has been noted to respond similarly to pediatric RMS. Our patient would be considered Stage II (cT2aN0M0), with tumor extension into an unfavorable site (orbital apex) and FOX01 translocation positive, which would correspond to the intermediate risk group of 50-70% event-free survival [10].

Regarding prognostication and survival of HNRMS, the reported 5-year overall survival (OS) ranges from 33% to 45% [8]. Wu et al. [8] reported their single-institution findings of 59 adult HNRMS patients and reported a five-year OS rate of 36%, with metastasis to cervical lymph nodes in 29% of patients. Worse prognostication was associated with tumor size greater than five centimeters, positive surgical margins, and cervical lymph node involvement. Local recurrence and distant metastasis were the primary causes of treatment failure [8,9]. The extent of disease (localized vs. regional) is a key prognostic factor of HNRMS relative survival (RS) more than involvement of a primary site, with regional disease portending a worse prognosis [1,2].

The parameningeal subsite of HNRMS is also associated with a poorer prognosis and earlier recurrence. This is likely because these tumors tend to extend into meningeal and intracranial sites, as seen in our patient [7]. In a database review of 186 adult patients with sinonasal/parameningeal RMS, Stepan et al. found alveolar to be the most common histologic type, comprising 66.7% of adult cases [7]. Alveolar RMS was not shown to have a poorer prognosis than embryonal RMS within the sinonasal sites; however, increased age was correlated with a poorer prognosis regardless of type, with five-year survival rates of 31.9% in those aged 18-35 and 24.4% in patients older than 35 years [7].

In general, the treatment guidelines for adults are similar to those for children due to limited data available, which consists primarily of chemotherapy with the addition of radiation and/or surgical resection [10-13]. Although there are limited data for adults, surgical resection of parameningeal RMS in the pediatric population is correlated with a higher five-year survival rate [12]. Pediatric RMS responds well to induction chemotherapy with concurrent chemoradiotherapy, but adult RMS tends to be more aggressive with a worse prognosis. Although initial surgical resection may be difficult secondary to the anatomic location of the lesion, the use of induction chemotherapy to shrink the tumor can allow for surgical resection in adult patients [14]. Kobayashi et al. reported treatment outcomes of 37 HNRMS patients undergoing either delayed primary excision after induction chemotherapy versus concurrent chemoradiotherapy after induction. In patients with a good response to induction chemotherapy, the surgical excision group had better three-year locoregional control than the chemoradiotherapy group [14].

While there was no direct extension into the cranial fossa in our patient, there was the involvement of the PPF and orbital apex. With the patient opposed to an orbital exenteration, a pterygopalatine fossa dissection was a feasible option. Our patient underwent this procedure without any orbital injury, cranial nerve injury, or CSF leak. Craniofacial approaches have been reported, but these result in transfacial incisions or facial osteotomies, which can be disfiguring and have an increased risk of neural and vascular injury secondary to poorer visualization [15-17].

## Conclusions

We presented a 46-year-old male with a T2aN0M0 parameningeal alveolar RMS with involvement of the orbital apex, abutment of the planum sphenoidale, and extension to the FR who underwent IC followed by endoscopic resection and adjuvant concurrent chemotherapy with proton beam radiation. The treatment course was modeled after pediatric protocols for similar parameningeal RMS. Currently, the patient is 18 months disease-free without significant comorbidities or side effects from their treatment. To the authors’ knowledge, there are no previous reports of this type of operative and post-operative treatment regimen for parameningeal RMS in an adult, specifically with adult endoscopic resection with proton beam therapy.
